# The presence of disulfide bonds reveals an evolutionarily conserved mechanism involved in mitochondrial protein translocase assembly

**DOI:** 10.1038/srep27484

**Published:** 2016-06-06

**Authors:** Lidia Wrobel, Anna M. Sokol, Magdalena Chojnacka, Agnieszka Chacinska

**Affiliations:** 1 International Institute of Molecular and Cell Biology, 02-109 Warsaw, Poland; 2 Department of Microbiology, Monash University, Melbourne, Victoria 3800, Australia

## Abstract

Disulfide bond formation is crucial for the biogenesis and structure of many proteins that are localized in the intermembrane space of mitochondria. The importance of disulfide bond formation within mitochondrial proteins was extended beyond soluble intermembrane space proteins. Tim22, a membrane protein and core component of the mitochondrial translocase TIM22, forms an intramolecular disulfide bond in yeast. Tim22 belongs to the Tim17/Tim22/Tim23 family of protein translocases. Here, we present evidence of the high evolutionary conservation of disulfide bond formation in Tim17 and Tim22 among fungi and metazoa. Topological models are proposed that include the location of disulfide bonds relative to the predicted transmembrane regions. Yeast and human Tim22 variants that are not oxidized do not properly integrate into the membrane complex. Moreover, the lack of Tim17 oxidation disrupts the TIM23 translocase complex. This underlines the importance of disulfide bond formation for mature translocase assembly through membrane stabilization of weak transmembrane domains.

Disulfide bonds are covalent links between two cysteine residues of proteins, typically introduced posttranslationally through thiol-disulfide exchange reactions. Disulfide bond formation can be crucial for the correct folding of a protein, maintenance of protein structure, and regulation of its redox-dependent functions. Most proteins that contain intramolecular disulfide bonds perform their function as secreted proteins in the extracellular milieu. However, a substantial group of proteins that require disulfide bonds is present in the intermembrane space (IMS) compartment of mitochondria. The majority of the IMS proteome are proteins that contain characteristic twin cysteine motifs, arranged as CX_3_C or CX_9_C, in their amino acid sequence^1^,^2^,^3^,^4^. The import and oxidative folding of such precursor proteins are mediated by the mitochondrial IMS assembly (MIA) pathway^5^,^6^. Representatives of classic substrates of the MIA pathway include small Tim chaperones, such as Tim9 or Tim10, and proteins that are involved in the proper function and assembly of respiratory chain complexes, including Cox12, Cox17, and Cox19. The activity of this pathway depends on two essential components, oxidoreductase Mia40 and sulfhydryl oxidase Erv1^2^,^3^,^7^. The mechanisms that are utilized by the MIA pathway are unique as the oxidation of incoming precursor proteins provides a means to trap them in the IMS and prevent their escape^8^,^9^.

Redox reactions are also important during the biogenesis of atypical IMS proteins. One example is the IMS protease Atp23, which is a heavily oxidized protein that is peripherally associated with the inner mitochondrial membrane. The import and maturation of Atp23 were found to be assisted by Mia40^10^. In addition, Mia40 catalyzes the formation of disulfide bonds in anamorsin, a protein that has been implicated in Fe/S cluster assembly^11^. Beside the crucial role in mitochondrial protein structure, cysteine residues oxidation is important in the regulation of protein function within mitochondria. The conserved cysteine residues of the mitochondrial ADP/ATP carrier were shown to be important for its maturation^12^. Recently, Mia40-mediated formation of an intramolecular disulfide bond between the IMS proteins MICU1 and MICU2 was shown to be critical for the control of mitochondrial Ca^2+^ uniporter function in human cells^13^. The formation of the disulfide bond can also be part of a protein quality control system. The presence of the disulfide bond within the structure of the mitochondrial ribosomal protein Mrp10 prevents its proteolytic degradation in the IMS prior to mitochondrial matrix translocation^14^. Thus, the importance of disulfide bond formation in soluble proteins is well defined^2^,^3^,^7^. Recent work revealed that membrane protein Tim22, the core component of the TIM22 protein translocase complex responsible for the import of multispanning membrane proteins in the yeast *Saccharomyces cerevisiae*^15^, was found in the oxidized state^16^,^17^. The oxidation of Tim22 during import supports its proper membrane integration and assembly of the mature TIM22 complex^16^,^17^. Furthermore, this oxidation is facilitated by a direct interaction between Mia40 and Tim22 during its biogenesis^16^.

The role of cysteine oxidation in the biogenesis of the yeast multi-spanning membrane protein Tim22 led us to systematically determine the presence of this modification in the Tim17/Tim22/Tim23 family. Moreover, we were interested in disulfide bond conservation within eukaryotes and the presence of this modification relative to membrane topology. We demonstrated a conserved pattern of cysteine residues in Tim17 and Tim22 but not Tim23. Our analysis revealed the formation of disulfide bonds in Tim17 and Tim22 proteins in *S. cerevisiae*, *C. albicans*, and *H. sapiens*. Moreover, we provided membrane topology models for Tim17 and Tim22, including the positioning of disulfide bond(s). Thus, oxidation of Tim17 and Tim22, which promotes translocase complex biogenesis, is a highly conserved feature across eukaryotes.

## Results and Discussion

### Oxidation of Tim17 in *S. cerevisiae*

**Figure 1:**
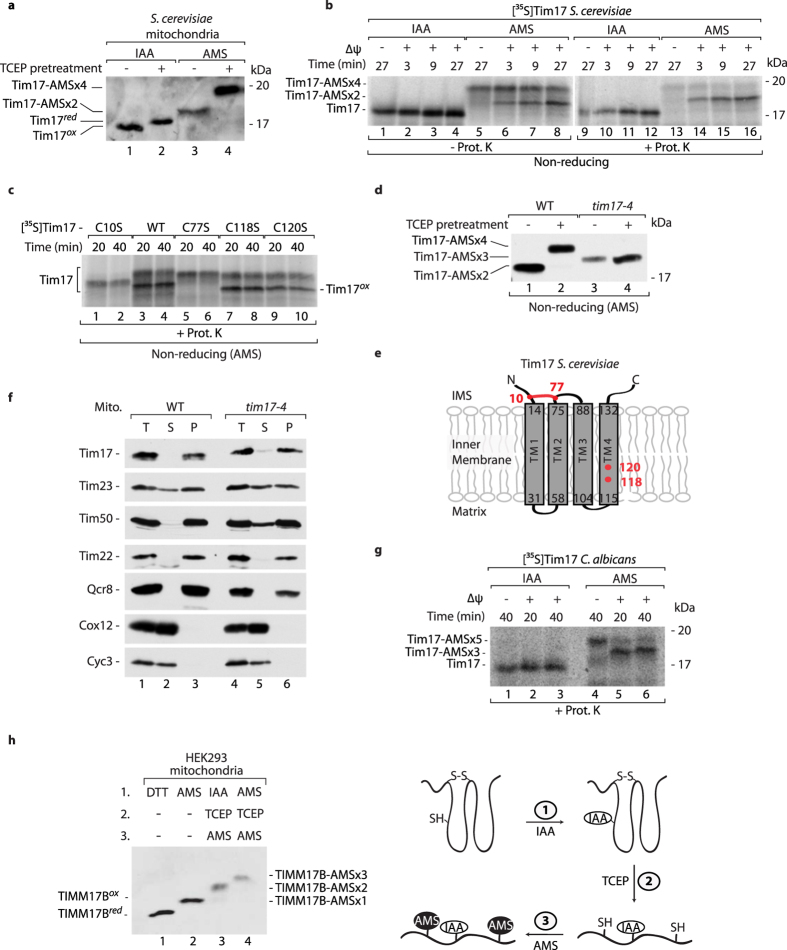
Tim17 forms an intramolecular disulfide bond in fungal and metazoan mitochondria. (**a**) Mitochondria were isolated from wildtype *S. cerevisiae* and pretreated with TCEP prior to denaturation in Laemmli buffer with 50 mM IAA or 10 mM AMS. (**b,c**) Radiolabeled *S. cerevisiae* Tim17 precursors were incubated with mitochondria isolated from wildtype *S. cerevisiae* in the presence or absence of inner membrane electrochemical potential when indicated. (**d**) Mitochondria were isolated from wildtype and *tim17-4 S. cerevisiae* and pretreated with TCEP prior to denaturation in Laemmli buffer with 10 mM AMS. (**e**) The amino acid sequence of Tim17 (UniProt: P39515) was analyzed by the transmembrane predictor program hmmtop. The red dots indicate cysteine residues. The red line indicates disulfide bond. (**f**) Mitochondria isolated from WT and *tim17-4* strain were subjected to alkaline carbonate extraction. T, total mitochondrial protein extract; S, supernatant; P, pellet of mitochondrial membranes. (**g**) Radiolabeled *C. albicans* Tim17 precursor was incubated with wildtype mitochondria of *C. albicans*. (**b,c,g**) The import reaction was stopped on ice and by adding 10 mM AMS or 50 mM IAA. Excess of nonimported precursor was removed by proteinase K. Samples were analyzed under non-reducing conditions by SDS-PAGE followed by autoradiography. (**h**) Left panel: Mitochondria were isolated from HEK293 cells and treated either with 100 mM DTT (lane 1) or with 15 mM AMS (lane 2). An indirect thiol trapping assay was performed to identify the presence of disulfide bonds. Free cysteine groups were modified by 50 mM IAA or 15 mM AMS, and disulfide bonds were then reduced with 10 mM TCEP to yield free cysteine residues that were further modified with 15 mM AMS (lanes 3 and 4) Right panel: Model of human TIMM17 redox state. The free cysteine was first modified by IAA. The disulfide bond was then reduced with TCEP to yield free cysteine residues that were further modified by AMS. (**a,d,f,h**) The samples were analyzed by SDS-PAGE and Western blot. red, reduced; ox, oxidized; ΔΨ, inner membrane electrochemical potential; IMS, intermembrane space; WT, wildtype.

Tim17, Tim22, and Tim23 are the essential core components of mitochondrial inner membrane translocation machineries. Tim22 from *Saccharomyces cerevisiae* yeast was shown to form an intramolecular disulfide bond^16^,^17^. Thus, we sought to determine the redox state of Tim17. *S. cerevisiae* Tim17 contains four cysteine residues. We used a thiol-modification assay, in which free sulfhydryl groups can be irreversible modified with either iodoacetmide (IAA) or 4-acetamido-4′-maleimidylstilbene-2,2′-disulfonic acid (AMS). The latter thiol-modifying agent binds to a free sulfhydryl group, providing a 500 Da size shift in protein mass and resulting in a change in migration. Isolated mitochondria were pretreated with tris(2-carboxyethyl)phosphine (TCEP) to reduce disulfide bonds if indicated (Fig. 1a, lanes 2 and 4). Tim17 was analyzed in the presence of IAA and found to migrate faster on the gel in the sample not treated with TCEP compared with the pretreated sample (Fig. 1a; compare lanes 1 and 2). This suggests that Tim17 contained oxidized cysteine residues that were reduced by TCEP and thus liberated to bind IAA. However, Tim17 has four cysteine residues, so either one or two disulfide bonds could be formed. In the samples that were directly incubated with AMS, a slowdown in Tim17 migration was observed compared with the samples that were treated with IAA (Fig. 1a, compare lanes 1 and 3). This indicates that Tim17 also contains cysteine residues in the reduced state, which are available for modification with AMS without prior reduction. These results demonstrate that *S. cerevisiae* Tim17 is a partially oxidized protein that likely has one disulfide bond and two cysteine residues with free thiol groups.

We imported radiolabeled *S. cerevisiae* Tim17 into isolated mitochondria and observed efficient generation of the oxidized form of Tim17, which was present inside mitochondria in the protease K-protected location (Fig. 1b, lanes 9–16). A recent study showed that Tim22 import relies on the presence of an electrochemical inner membrane potential^16^. This was also valid for the oxidation of Tim17 (Fig. 1b, lanes 5, 13). To determine which cysteine residues form the disulfide bond, we synthesized radiolabeled Tim17 precursors in which single cysteine residues were substituted for serine (Tim17-C10S, Tim17-C77S, Tim17-C118S, Tim17-C120S). We analyzed the import of Tim17 variants and compared their migration with wildtype Tim17 (Fig. 1c; Supplementary Fig. S1a). A different migration pattern of Tim17-C10S and Tim17-C77S was observed in both, the samples treated (Fig. 1c) or not (Supplementary Fig. S1a) with proteinase K, indicating that this migration pattern is not a consequence of protease activity. In the case of Tim17-C10S and Tim17-C77S we did not detect any oxidized form, despite differential migration (Fig. 1c, lanes 1–2 and 5–6). Thus, Cys10 and Cys77 are necessary for Tim17 oxidation. To support our import data, we analyzed the redox state of Tim17 in the *tim17-4* strain, in which Cys10 was substituted for Arg^18^,^19^. As previously reported^18^,^19^, the cells with Tim17-4 expressed a strong growth defect at the higher temperature (Supplementary Fig. S1b). Mitochondria that were isolated from *tim17-4* and wildtype cells were pretreated with TCEP to reduce existing disulfide bonds (Fig. 1d, lanes 2, 4). Subsequently, the samples were modified by AMS. Comparison of the migration pattern between the wildtype and *tim17-4* strains indicated that mutated Tim17 that lacked Cys10 was present in a reduced form (Fig. 1d).

The structure of the TIM23 translocase complex is unknown. Topology prediction for yeast Tim17 showed that Cys residues 10 and 77 are on the IMS side of the inner mitochondrial membrane (Fig. 1e). To provide experimental evidence for this arrangement of Tim17, we decided to introduce the Myc-tag at the N-terminus of Tim17. To address the membrane integrity of _Myc_Tim17 we applied the carbonate extraction assay. The _Myc_Tim17 variant was found in the membrane pellet, together with a membrane protein Qcr8, indicating _Myc_Tim17 integration into the mitochondrial membrane (Supplementary Fig. S1c). The matrix protein Mdh1 was found only in the soluble fraction, confirming the assay specificity (Supplementary Fig. S1c). We performed the protease accessibility assay upon outer mitochondrial membrane rupture (mitoplasting) and concluded that the N-terminus of Tim17 is present on the IMS side of the inner membrane (Supplementary Fig. S1d), topologically permitting disulfide bond formation between Cys10 and Cys77 of Tim17.

To address the importance of Tim17 oxidation, we analyzed the membrane integrity of Tim17, and other components of the TIM23 complex, in the *tim17-4* cells (Fig. 1f). The Tim17-4 mutant protein was found in the membrane pellet, indicating its proper integration into the membrane (Fig. 1f). However, the other components of the TIM23 translocase, such as Tim23 or Tim50, were partially extracted from the *tim17-4* mitochondrial membranes, in contrast to wildtype (Fig. 1f, compare lanes 2 and 5). Thus, the lack of Tim17 oxidation destabilizes the TIM23 complex.

### The disulfide bond is present in fungal and metazoan Tim17, but not in Tim23

Sequence alignment of Tim17 from fungi and metazoa indicated a strong conservation of two Cys residues (Supplementary Fig. S2)^20^. This observation raised the possibility that Cys residues are engaged in disulfide bond formation in other organisms. We imported radiolabeled *Candida albicans* Tim17 into isolated mitochondria. *C. albicans* Tim17 contains five Cys residues (Supplementary Fig. S2). The partial oxidation of Tim17, corresponding to two Cys residues, was observed in the assay with the thiol-modifying agent (Fig. 1g, lanes 5–6). This oxidation of Tim17 was dependent on the electrochemical inner membrane potential (Fig. 1g, lane 4). Thus, the *C. albicans* Tim17 protein is oxidized upon import into the mitochondria isolated from *C. albicans*.

Mammals have two homologs of yeast Tim17: TIMM17A and TIMM17B. In mammalian cells, both *TIMM17A* and *TIMM17B* are ubiquitously expressed, and they are nearly identical in their amino acid sequence. However, TIMM17B appears to play a more fundamental housekeeping role in mitochondrial biogenesis^21^,^22^. We applied a thiol trapping assay to protein extracts from human embryonic kidney 293 (HEK293) cells and analyzed the samples using antibodies that were directed against human TIMM17B (Fig. 1h). In this assay, free sulfhydryl groups were first irreversibly blocked with IAA or AMS prior to existing disulfide bond/s reduction. As a result of reduction, sulfhydryl groups were available for subsequent modification by AMS (Fig. 1h; lanes 3, 4). Human TIMM17B possesses three cysteine residues in its amino acid sequence. The gel pattern (Fig. 1h) led us to conclude that TIMM17B forms one disulfide bond in HEK293 cells and possesses one free sulfhydryl group (Fig. 1h). In summary, we found that Tim17 is present in an oxidized state in *S. cerevisiae*, *C. albicans*, and *H. sapiens* mitochondria. Altogether, formation of the disulfide bond within the Tim17 structure between two cysteine residues is a conserved feature among eukaryotes.

The *S. cerevisiae* and human Tim23 possess two cysteine residues in the amino acid sequence. We assessed the redox state of Tim23 in mitochondria isolated from *S. cerevisieae* cells (Supplementary Fig. S3a) and human HEK293 cells (Supplementary Fig. S3b). The migration of Tim23 in the samples that were untreated *vs*. pretreated with reducing reagent indicated that Tim23 is present in a reduced state (Supplementary Fig. S3a, b). In support of this finding, the cysteine residues of Tim23 were found not to be conserved between various organisms (data not shown). Thus, consistent with weak cysteine residue conservation, Tim23 is present in a reduced state in fungi and metazoa.

### Involvement of the MIA pathway in Tim17 oxidation

**Figure 2:**
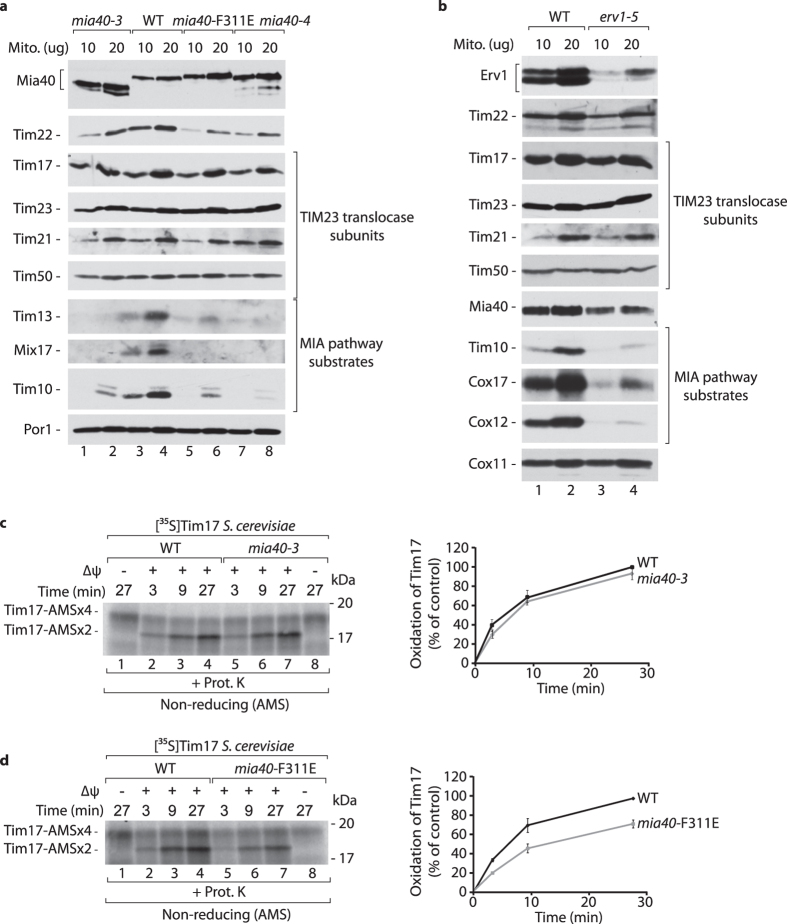
The MIA pathway is involved in the Tim17 biogenesis. (**a,b**) Mitochondria were isolated from wildtype, *mia40-3*, *mia40-4*, *mia40*-F311E and *erv1-5* mutant strains grown on YPG liquid medium at 19 °C and shifted for 6 h to 37 °C. (**c,d**) Radiolabeled Tim17 precursor was incubated with mitochondria isolated from wildtype and *mia40-3* (**c**) or wildtype and *mia40*-F311E (**d**) mutant strains grown at 19 °C. Nonimported protein was removed by proteinase K and samples were analysed under non-reducing conditions in the presence of 10 mM AMS followed by autoradiography. Right panels: quantification of radiolabeled Tim17 import. Mean +/− S.E.M, n = 3. ΔΨ, inner membrane electrochemical potential; WT, wildtype.

We analyzed the involvement of the MIA pathway components, oxidoreductase Mia40 and sulfhydryl oxidase Erv1^2^,^3^,^5^,^7^,^8^,^23^, in the import of Tim17. Firstly, we determined the levels of Tim17 in the mitochondria isolated from the temperature sensitive *mia40* mutant strains (*mia40-3*, *mia40-4*, *mia40-*F311E)^5^,^16^,^23^,^24^,^25^, which were cultured under restrictive temperature conditions (Fig. 2a). In agreement with previously published data^16^, we observed the decrease in the levels of Tim22 together with classic MIA substrates, but the levels of Tim17 remained equal in the *mia40* mutant mitochondria (Fig. 2a). The levels of Tim17 were also equal in mitochondria isolated from the *erv1-5* mutant strain (Fig. 2b)^8^,^23^.

To kinetically characterize the Tim17 biogenesis requirement for Mia40, we imported radiolabeled Tim17 precursor into the mitochondria isolated from *mia40-3* (Fig. 2c) and *mia40*-F311E (Fig. 2d) strains. The efficiency of Tim17 import and oxidation was decreased only in the case of mitochondria with Mia40-F311E (Fig. 2d). This observation can be explained by the decreased ability of Mia40-F311E to bind its substrates via hydrophobic interactions^16^,^25^. Mitochondria isolated from the *erv1-5* mutant were also impaired in the import of Tim17 precursor (Supplementary Fig. S3c). Thus, the Tim17 oxidative biogenesis relies on the activity of the MIA pathway components.

### Membrane topology of Tim22

**Figure 3:**
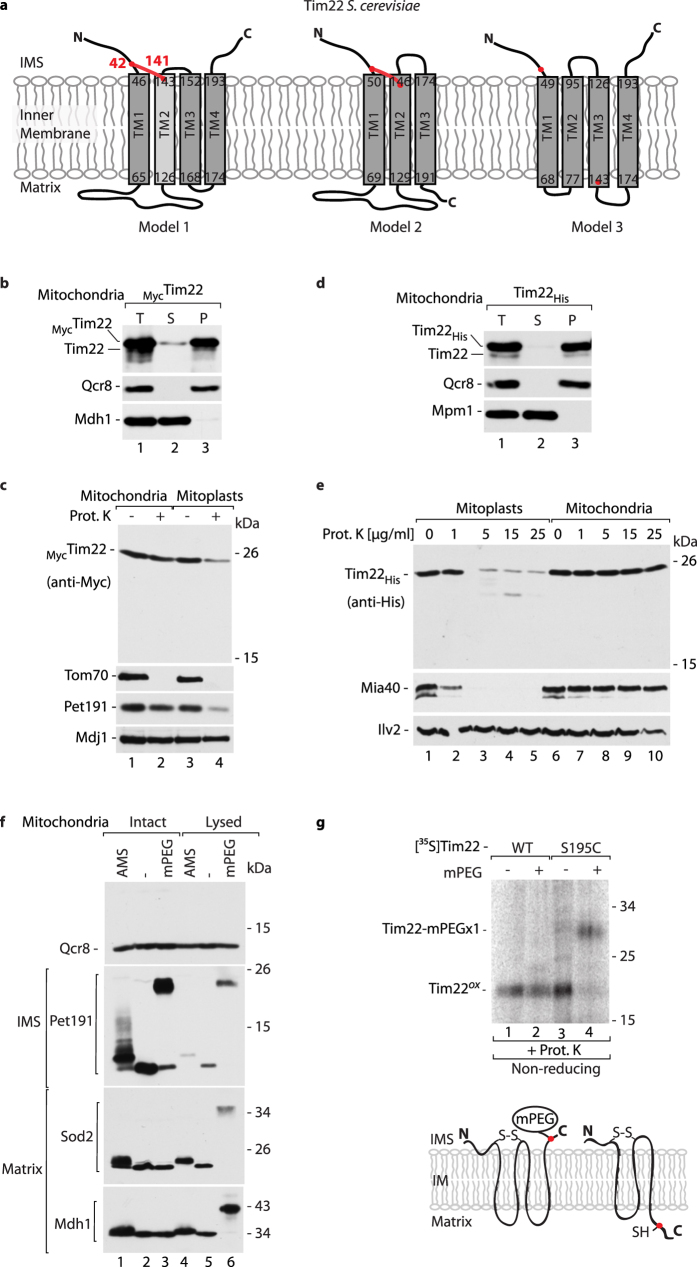
Transmembrane topology of *S. cerevisiae* Tim22. (**a**) The amino acid sequence of Tim22 (UniProt: Q12328) was analyzed by transmembrane predictor programs. Model 1 by Phobius: four transmembrane domains with TM2 predicted as less hydrophobic (no significance) marked in light grey. Model 2 by TMPred. Model 3 by hmmtop. The red dots indicate the presence of a cysteine residue. The red lines indicate the presence of a possible disulfide bond. (**b,d**) Mitochondria isolated from wildtype and strains overexpressing _Myc_Tim22 (**b**) and Tim22_His_ (**d**) were subjected to alkaline carbonate extraction. T, total mitochondrial protein extract; S, supernatant; P, pellet of mitochondrial membranes. (**c,e**) Mitochondria isolated from strains expressing _Myc_Tim22 (**c**) and Tim22_His_ (**e**) were incubated in hypotonic buffer for 30 minutes to generate mitoplasts, when indicated. Mitoplasts and intact mitochondria were treated with 50 μg/ml proteinase K, when indicated. (**f**) Intact or lysed mitochondria were incubated with 10 mM AMS or 2 mM mPEG_5_ for 30 min at 25 °C. The samples of lysed mitochondria were precipitated with 10% TCA. (**g**) Radiolabeled Tim22 wildtype or Tim22-S195C precursor were imported into isolated mitochondria, followed by proteinase K digestion of nonimported precursors. The mitochondria were further incubated with 2 mM mPEG_5_ for 30 min at 25 °C. Samples were denatured under non-reducing conditions in the presence of 50 mM IAA. Lower panel: Possible scenarios of mPEG_5_ modification according to the transmembrane topology of Tim22. (**b–g**) Samples were analyzed by SDS-PAGE followed by Western blot or autoradiography. ox, oxidized; IMS, intermembrane space; IM, inner membrane; WT, wildtype.

Because of the lack of structural data concerning the TIM22 translocase complex, both the topology as well as topological positioning of the disulfide bond in Tim22 have remained unclear. Furthermore, the positioning of transmembrane segments in Tim22 is equivocal. According to bioinformatics transmembrane topology prediction analyses, three different models of Tim22 topology are possible (Fig. 3a). The first and third model indicate that Tim22 possesses four transmembrane segments, in contrast to the second model that predicts that Tim22 has only three transmembrane segments. In the case of the third model, the disulfide bond cannot be formed because the cysteine residues are located on opposite sides of the inner membrane (Fig. 3a, model 3). Thus, the third model is likely to be incorrect. To distinguish between the first and second model (Fig. 3a), additional biochemical experiments were performed. Firstly, to determine the orientation of the first transmembrane domain we introduced the Myc-tag on the N-terminus of Tim22. _Myc_Tim22 was properly integrated into the mitochondrial membrane, similarly to a control membrane protein Qcr8, whereas the matrix Mdh1 was found in the soluble fraction (Fig. 3b). Upon mitochondrial swelling (mitoplasting) the N-terminal part of _Myc_Tim22 was digested by proteinase K, indicating its localization on the IMS side of the inner membrane (Fig. 3c). According to the models, the C-terminal part of Tim22 can localize either to the IMS (model 1) or to the matrix (model 2). To discriminate the position of the C-terminus between either the IMS or the matrix, we introduced the His-tag on the C-terminus of Tim22. Tim22_His_ was also properly integrated into the mitochondrial membrane (Fig. 3d). The protease accessibility assay on mitoplasts revealed that the C-terminus of Tim22 is present on the IMS side of the IM as it was digested by the increasing concentration of proteinase K (Fig. 3e). These results suggest that both termini of Tim22 are present on the IMS side.

To further support our findings, we decided to use a thiol-modifying agent that does not cross the inner mitochondrial membrane. We used methoxypolyethylene glycol maleimide (mPEG_5_), which increases the protein mass by 5 kDa per one cysteine residue. To test the permeability of the inner mitochondrial membrane, the mitochondria were incubated with either AMS or mPEG_5_, and mitochondrial proteins were detected with specific antibodies (Fig. 3f). Qcr8 was not modified because it lacks cysteine residues, indicating the specificity of the assay. Pet191 is a soluble IMS protein with one free cysteine residue, which was efficiently modified by AMS and mPEG_5_. In contrast, the matrix proteins Sod2 and Mdh1, which both possess one free cysteine residue, were modified only by AMS and not by mPEG_5_ in intact mitochondria (Fig. 3f). However, upon mitochondrial lysis, Sod2 and Mdh1 were efficiently modified by mPEG_5_ (Fig. 3f; compare lanes 3 and 6). This indicates that the inner membrane in intact mitochondria is impermeable to mPEG_5_. Thus, mPEG_5_ can be used to assess the location of the C-terminus of Tim22. An additional cysteine residue, which could be a potential target for mPEG_5_ modification, was introduced at position 195 of the Tim22 amino acid sequence, close to the C-terminal end. Radiolabeled wildtype Tim22 and Tim22-S195C precursor proteins were imported into wildtype mitochondria (Fig. 3g). The modification of Tim22-S195C with mPEG_5_ resulted in a prominent shift in its migration, which was not observed in the case of wildtype Tim22. This shift indicates that the C-terminus of Tim22-S195C is available for modification. We evaluated the integrity of imported Tim22-S195C that was modified with mPEG_5_ by using the carbonate extraction assay (Supplementary Fig. S3d). We found that wildtype and modified Tim22-S195C were fully integrated into the mitochondrial membrane (Supplementary Fig. S3d). These results favor the first model (Fig. 3a, model 1), in which *S. cerevisiae* Tim22 possesses four transmembrane regions, and a disulfide bond is present on the IMS side of the inner membrane.

### Complex oxidation pattern of Tim22 acquired in evolution

**Figure 4:**
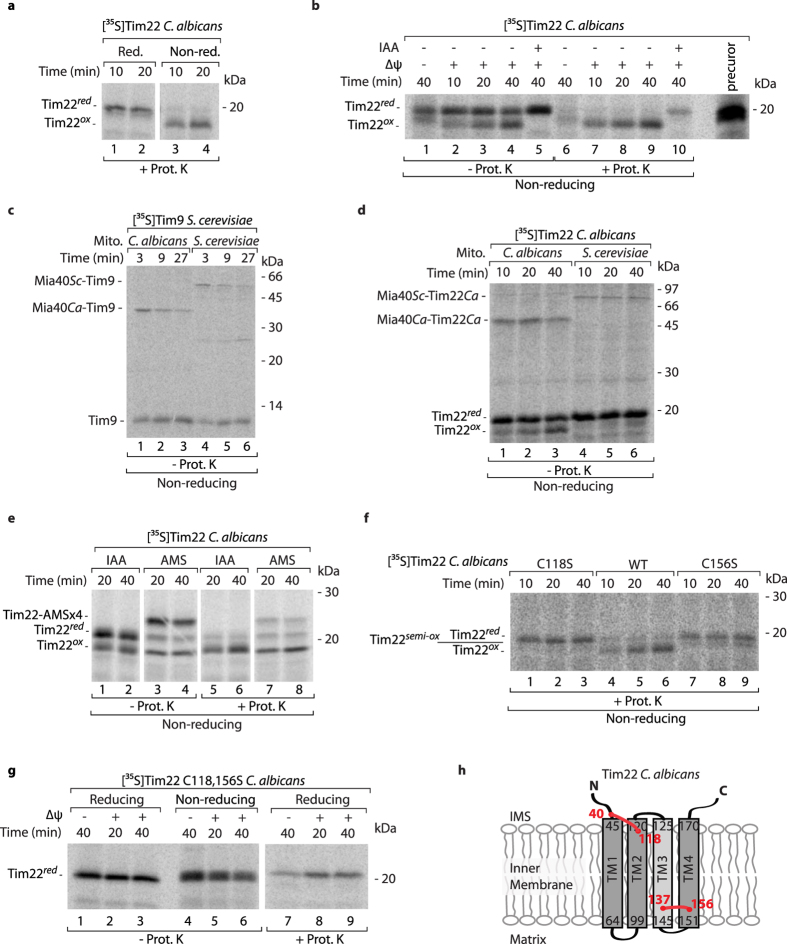
*C. albicans* Tim22 interacts with Mia40 and becomes oxidized. (**a,b**) Radiolabeled *C. albicans* Tim22 was incubated with mitochondria isolated from wildtype *C. albicans*. Nonimported protein was removed by proteinase K, when indicated. (**b**) When indicated, the inner membrane potential was dissipated, or free cysteine residues were blocked with iodoacetamide (IAA) prior to the import reaction. Precursor, 13% of the whole import reaction input. (**c,d**) Radiolabeled *S. cerevisiae* Tim9 or *C. albicans* Tim22 were incubated with mitochondria isolated from either wildtype *S. cerevisiae* or wildtype *C. albicans.* (**e**) Radiolabeled *C. albicans* Tim22 was incubated with mitochondria isolated from wildtype *C. albicans*. Nonimported protein was removed by proteinase K, when indicated. The import reaction was stopped by transferring the samples on ice and adding 10 mM AMS or 50 mM IAA. (**f,g**) Radiolabeled mutant versions of *C. albicans* Tim22 (Tim22-C118S, Tim22-C156S, and Tim22-C118,156S) were incubated with mitochondria isolated from wildtype *C. albicans*. (**a–g**) The samples were analyzed under reducing or non-reducing conditions as indicated by SDS-PAGE followed by autoradiography. (**h**) The amino acid sequence of *C. albicans* Tim22 (Uniprot:Q59KG4) was analyzed by the transmembrane predictor program Phobius with TM3 predicted as less hydrophobic (no significance) marked in light grey. The red lines indicate the presence of disulfide bonds. red, reduced; ox, oxidized; semi-ox, semi-oxidized; ΔΨ, inner membrane electrochemical potential; IMS, intermembrane space; WT, wildtype.

We noticed that other fungi possess one more couple of conserved cysteine residues in addition to the disulfide bond forming cysteine residues of *S. cerevisiae* (Supplementary Fig. S4a). *Candida albicans* is a representative of fungi, in which four cysteine residues are present within the Tim22 sequence (Supplementary Fig. S4a). To determine the redox state of Tim22 in *C. albicans*, we imported radiolabeled Tim22 into mitochondria that were isolated from *C. albicans* (Fig. 4a). Differential migration between Tim22 that was analyzed under reducing and non-reducing conditions could be attributed to the oxidized state of Tim22. Time-dependent generation of the oxidized form of Tim22 that is present inside mitochondria, and protected from proteinase K, was observed during the import of radiolabeled Tim22 into isolated mitochondria (Fig. 4b, lanes 7–9). Similarly to yeast Tim22 and Tim17 (Fig. 1b)^16^, the import and oxidation of *C. albicans* Tim22 was inhibited when the inner mitochondrial membrane potential was dissipated (Fig. 4b, lanes 1, 6). The oxidation of cysteine residues can be irreversibly blocked by IAA. *C. albicans* Tim22 oxidation was blocked, and import into the proteinase K-protected location inside mitochondria was strongly inhibited upon IAA modification (Fig. 4b, lanes 5, 10).

The import and oxidation of *S. cerevisiae* Tim22 was shown to be accompanied by the interaction with the oxidoreductase Mia40^16^. The dependence of Tim22 on Mia40 was questioned^17^. To assess the involvement of Mia40 in the import of *C. albicans* Tim22, we isolated mitochondria from both *S. cerevisiae* and *C. albicans* and first incubated them with Tim9, the classic MIA pathway substrate (Fig. 4c). Tim9 efficiently formed an intermediate with *S. cerevisiae* Mia40 (Mia40*Sc*) and *C. albicans* Mia40 (Mia40*Ca*). The Mia40*Ca*-Tim9 intermediate migrated faster on the gel compared with the Mia40*Sc*-Tim9 intermediate because of the lower molecular weight of Mia40 in *C. albicans* (27.9 kDa). Next, we imported radiolabeled *C. albicans* Tim22 and observed the formation of Mia40-Tim22 intermediates that resembled the Mia40-linked intermediates of Tim9 (Fig. 4d). *C. albicans* Tim22 became oxidized in mitochondria that were isolated from *C. albicans*, but was unable to oxidize upon import into *S. cerevisiae* mitochondria (Fig. 4d, lanes 1–3 and 4–6). Thus, the Mia40 binding during Tim22 biogenesis is commonly observed in fungi.

The *C. albicans* Tim22 protein has four cysteine residues within its sequence. Therefore, oxidized Tim22 can potentially form two disulfide bonds. To verify this hypothesis, we imported radiolabeled Tim22 into mitochondria and analyzed the samples in the presence of IAA or AMS (Fig. 4e). The Tim22 precursor outside mitochondria is present in a reduced state. Therefore, all four cysteine residues were modified by AMS, resulting in a clear change in protein migration on the gel (Fig. 4e, lanes 3, 4). However, only oxidized Tim22, which was not modified by AMS, was present inside mitochondria (Fig. 4e, lanes 7, 8). Thus, *C. albicans* Tim22 forms two disulfide bonds upon import into mitochondria. To further support this notion, we imported radiolabeled Tim22 precursors that lacked either Cys118 or Cys156 (Fig. 4f). Neither of the precursors were able to fully oxidize inside mitochondria, but the formation of a weak band was observed in the case of the Tim22-C156S precursor, possibly indicating that it forms one disulfide bond (Tim22^*semi-ox*^; Fig. 4f, lanes 7–9). Furthermore, the import of the radiolabeled Tim22-C118,156S precursor that lacked both cysteine residues was analyzed under reducing and non-reducing conditions (Fig. 4g). No change in migration was observed, demonstrating that both cysteine residues are essential for the full oxidation of Tim22 (Fig. 4g). Based on these results and the transmembrane topology model of Tim22, we propose that Tim22 from *C. albicans* possesses two disulfide bonds. One disulfide bond is formed on the IMS side of Tim22 between Cys40 and Cys118, and the second one is formed on the matrix side between Cys137 and Cys156 (Fig. 4h).

**Figure 5:**
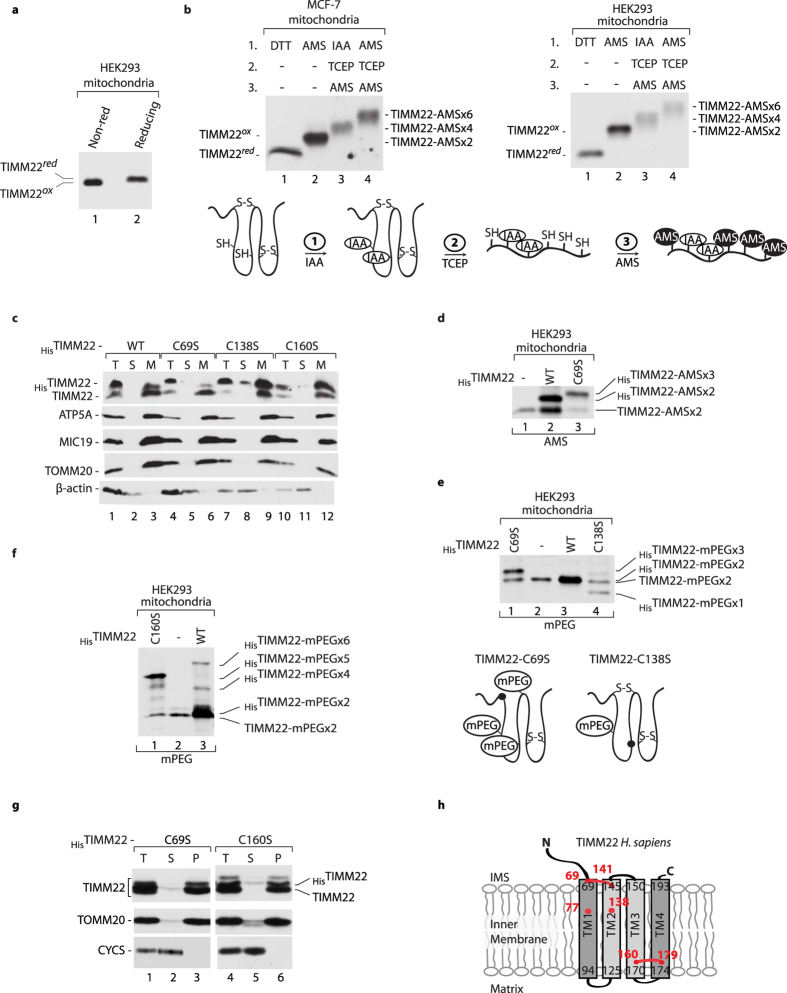
Human TIMM22 forms two disulfide bonds. (**a**) Mitochondria were isolated from HEK293 cells and analyzed under non-reducing or reducing conditions. (**b**) Upper panel: Mitochondria were isolated from MCF-7 or HEK293 cells and treated either with 100 mM DTT (lane 1) or with 15 mM AMS (lane 2). An indirect thiol trapping assay was performed to identify the presence of disulfide bonds. Free cysteine residues were modified by 50 mM IAA or 15 mM AMS (lanes 3 and 4), and disulfide bonds were then reduced with 10 mM TCEP to yield free cysteine residues that were further modified with 15 mM AMS (lanes 3 and 4). Bottom panel: Model of the human TIMM22 redox state. The two free cysteine residues were first modified by IAA. The disulfide bonds were then reduced with TCEP to yield four free cysteine residues that were further modified by AMS. (**c**) HEK293 cells that expressed _His_TIMM22-WT, _His_TIMM22-C69S, _His_TIMM22-C138S, and _His_TIMM22-C160S were analyzed for protein levels in total (T), post-mitochondrial supernatant (S), and mitochondrial (M) fractions. (**d**) Mitochondria were isolated from HEK293 cells that expressed _His_TIMM22-WT and _His_TIMM22-C69S. Free cysteine residues were modified with 15 mM AMS. (**e,f**) Mitochondria were isolated from HEK293 cells that expressed _His_TIMM22-WT, _His_TIMM22-C69S and _His_TIMM22-C138S (**e**) or _His_TIMM22-WT and _His_TIMM22-C160S (**f**). Free cysteine residues were modified with 10 mM mPEG_1.2_. (**e**) Lower panel: Model of the TIMM22 cysteine mutant redox state. _His_TIMM22-C69S is modified by three mPEG_1.2_ molecules and _His_TIMM22-C138S is modified by one mPEG_1.2_ molecule. (**g**) Mitochondria isolated from HEK293 cells expressing _His_TIMM22-C69S or _His_TIMM22-C160S were subjected to alkaline carbonate extraction. T, total mitochondrial protein extract; S, supernatant; P, pellet of mitochondrial membranes. (**a–g**) Samples were analyzed under reducing or non-reducing conditions by SDS-PAGE and Western blot. (**h**) The amino acid sequence of *H. sapiens* TIMM22 (UniProt: Q9Y584) was analyzed by the transmembrane predictor program Phobius with TM2 and TM3 predicted as less hydrophobic (nonsignificant) marked in light grey. The red dots indicate the presence of cysteine residue. The red lines indicate the presence of a possible disulfide bonds. red, reduced; ox, oxidized; IMS, intermembrane space; WT, wildtype.

In comparison to fungi, animals have gained even more conserved cysteine residues within the Tim22 sequence (Supplementary Fig. S4b). We analyzed the redox state of human TIMM22 in mitochondria that were isolated from HEK293 cells (Fig. 5a). Under non-reducing conditions, TIMM22 migrated slightly faster, which may indicate a change in redox state. Human TIMM22 has six cysteine residues (Supplementary Fig. S4b). To determine the number of disulfide bonds that are formed by TIMM22, mitochondria were isolated from two different human cell lines (HEK293 and MCF-7) and analyzed by thiol trapping assay (Fig. 5b). Comparisons of the migration patterns revealed that human TIMM22 forms two intramolecular disulfide bonds and has two free sulfhydryl groups (Fig. 5b, schema).

To evaluate which cysteine residues of TIMM22 are engaged in disulfide bond formation, we transfected cells with constructs that expressed His-tagged TIMM22 and its variants, in which cysteine residues were substituted for serine (_His_TIMM22-C69S, _His_TIMM22-C138S, _His_TIMM22-C160S). All variants were properly localized in the mitochondria, similar to the mitochondrial proteins ATP5A, MIC19, and TOMM20 (Fig. 5c). Wildtype TIMM22 protein possessed two free cysteine residues that were modified by AMS, providing an equal shift in migration for native TIMM22 and _His_TIMM22 (Fig. 5d; lanes 1, 2). _His_TIMM22-C69S was modified with three AMS molecules, indicating that the removal of Cys69 broke one disulfide bond in TIMM22 (Fig. 5d; lane 3). Thus, Cys69 is engaged in disulfide bond formation in TIMM22, which is consistent with the data obtained for *S. cerevisiae* and *C. albicans* (Figs 3a and 4h). Next, we analyzed mitochondria from cells that expressed _His_TIMM22-C138S and compared them with _His_TIMM22-C69S-expressing cells. We modified existing cysteine residues with the thiol-modifying agent methoxypolyethylene glycol maleimide (mPEG_1.2_), which provided an additional 1.2 kDa per one cysteine residue (Fig. 5e). TIMM22 and _His_TIMM22 were modified by two mPEG_1.2_ molecules (Fig. 5e, lanes 2, 3). Because of the breakage of one disulfide bond, _His_TIMM22-C69S was modified by three mPEG_1.2_ molecules (Fig. 5e, lane 1). However, _His_TIMM22-C138S was modified with only one mPEG_1.2_, suggesting that this mutant variant of TIMM22 possessed one free cysteine residue and two intact disulfide bonds (Fig. 5e, lane 4, schema). We also applied mPEG_1.2_ modification to the mitochondria isolated from HEK293 cells that expressed TIMM22-C160S. Interestingly, TIMM22-C160S was modified by five mPEG_1.2_ molecules, indicating that TIMM22-C160S is present in a fully reduced form in human mitochondria (Fig. 5f). To determine the importance of cysteine oxidation for TIMM22 membrane integration, we applied the carbonate extraction assay. TIMM22-C69S and TIMM22-C160S were properly inserted into the mitochondrial membrane (Fig. 5g). The membrane protein TOMM20 was found in the mitochondrial membranes, whereas the IMS protein CYCS was found in the soluble fraction, indicating that the carbonate extraction was successful (Fig. 5g). Altogether, the biochemical analyses and transmembrane prediction for human TIMM22 indicate that one disulfide bond could be formed on the IMS side of TIMM22 between Cys69 and Cys141, and another disulfide bond that joins the TM3 and TM4 is formed between Cys160 and Cys179 (Fig. 5h). The disulfide bond between Cys160 and Cys179 is prerequisite to form the disulfide bond between Cys69 and Cys141 in human TIMM22.

### Oxidized Tim22 promotes efficient assembly of the TIM22 translocase

**Figure 6:**
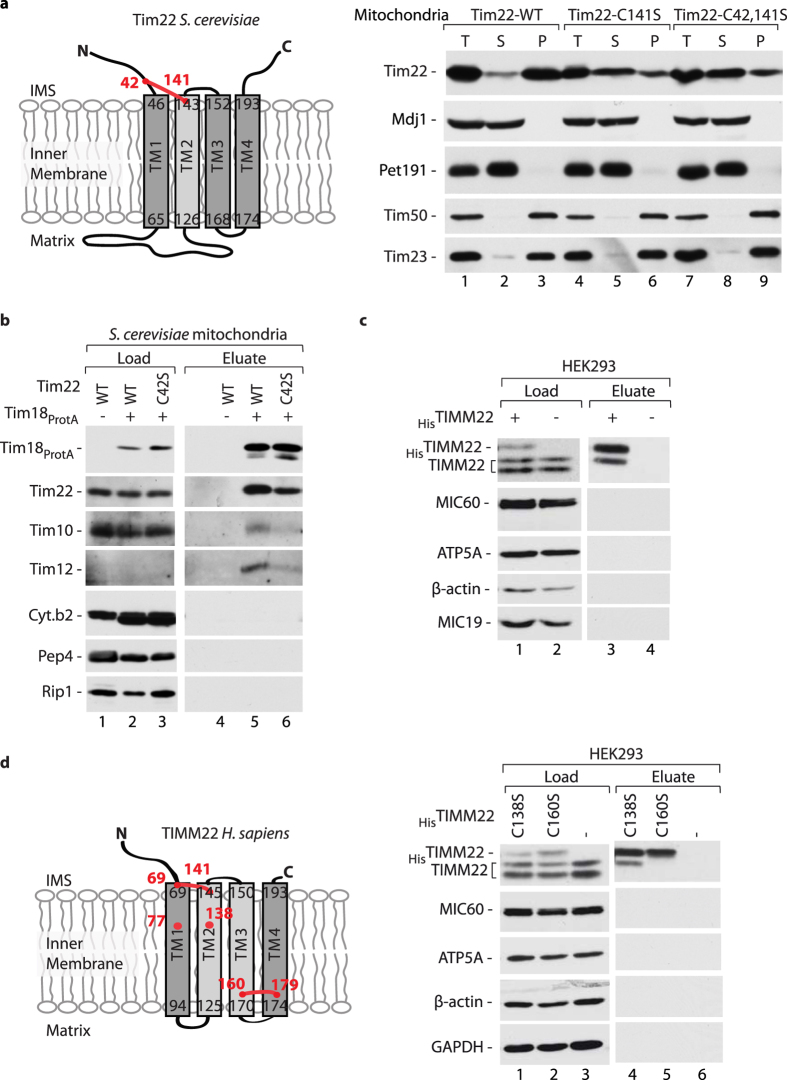
Tim22 disulfide bond formation is crucial for the TIM22 translocase assembly. (**a**) Mitochondria isolated from *S. cerevisiae* wildtype and *tim22* cysteine mutant strains were subjected to alkaline carbonate extraction. T, total mitochondrial protein extract; S, supernatant; P, pellet of mitochondrial membranes. (**b**) Mitochondria were isolated from *S. cerevisiae* wildtype or Tim18_ProtA_ strains that expressed either Tim22-WT or Tim22-C42S and solubilised in digitonin. (**c,d**) HEK293 cells that expressed _His_TIMM22-WT (**c**) or _His_TIMM22-C138S or _His_TIMM22-C160S (**d**) were solubilised in digitonin. (**b–d**) The protein complexes were isolated via affinity chromatography. Load: 3%; eluate: 100%. (**a–d**) Samples were analyzed under reducing conditions by SDS-PAGE and Western blot. IMS, intermembrane space; WT, wildtype.

The *S. cerevisiae tim22* mutants that lack disulfide forming Cys residues were viable and the levels of the TIM22 pathway substrates, carrier proteins such as Aac2 or Mir1, were not affected under basal growth conditions (Supplementary Fig. S5a, b)^16^,^17^. However, the lack of *S. cerevisiae* Tim22 cysteine residues impaired biogenesis of radiolabeled Tim22 during the *in organello* transport^16^,^17^. This underlines the role of disulfide bond formation in the dynamic processes of the integration and complex stability of the TIM22 translocase. Previously, we addressed the integrity of yeast Tim22 lacking a disulfide bond by using carbonate extraction method^16^. We found that in mitochondria isolated from yeast cultured at mild temperature, Tim22 cysteine-mutant variants were partially extracted from the membrane^16^. Here, we isolated mitochondria from strains expressing Tim22 cysteine-mutant variants grown under higher temperature (37 °C) conditions. The majority of Tim22 variants were found in the soluble fraction, in contrast to the wildtype Tim22, which was present almost exclusively in the membrane fraction (Fig. 6a). Thus, the Tim22 disulfide bond formation is important for the membrane integration/maintenance of Tim22. We examined the import of radiolabeled *tim22* cysteine mutant precursors under native conditions and found that the lack of oxidation impaired the Tim22 assembly into the matured TIM22 complex (Supplementary Fig. S5c)^16^,^17^. To further test this notion, mitochondria were isolated from yeast cells that expressed a tagged version of Tim18 together with either wildtype Tim22 or a form of Tim22 that lacked cysteine residue 42. These mitochondria were then subjected to the affinity purification assay (Fig. 6b). We did not observe any significant changes in the levels of Tim22 variants lacking Cys42 (Fig. 6b; load;^16^). We analyzed the TIM22 translocase subunits in the elution fraction. In the case of cells that expressed Tim22-C42S, we observed the decreased interaction between Tim18_ProtA_ and Tim22-C42S compared to wildtype Tim22 (Fig. 6b). Furthermore, in the background of Tim22-C42S, Tim10, and Tim12 were not stably attached to the TIM22 translocase (Fig. 6b). Thus, the lack of disulfide bond in Tim22 affects its membrane integration and causes a loss of TIM22 complex integrity in yeast. Similarly, the lack of disulfide bond formation in yeast Tim17 caused destabilization of the TIM23 translocase subunits, but in contrast to Tim22, reduced Tim17 was properly integrated into the mitochondrial membrane (Fig. 1f).

To assess the importance of the acquired disulfide bond linking Cys160 and Cys179 in human TIMM22, we expressed a His-tagged version of TIMM22 in human HEK293 cells and performed affinity purification from cellular protein extracts (Fig. 6c). Although TIMM22 (the core subunit of the TIM22 translocase) is conserved across species, its partner proteins that build the translocase are still unknown in humans^26^. However, we observed that _His_TIMM22 interacts with a native TIMM22 (Fig. 6c, lane 3), indicating the ability of _His_TIMM22 to correctly integrate into the inner mitochondrial membrane. We performed affinity purification of _His_TIMM22-C138S and _His_TIMM22-C160S from cellular protein extracts (Fig. 6d). A fraction of native TIMM22 was efficiently eluted together with the _His_TIMM22-C138S mutant, whereas the interaction between native TIMM22 and mutant _His_TIMM22-C160S was abolished (Fig. 6d, compare lanes 4 and 5). Thus, the lack of the second disulfide bond that is present in the human TIMM22 structure renders the protein unable to assemble into the mature TIM22 complex, despite its ability for membrane integration (Fig. 5g).

In summary, two highly conserved and essential core components of mitochondrial inner membrane translocases, Tim17 and Tim22, possess invariant cysteine residues that are oxidized. Our assays do not allow excluding other oxidative modifications. However, we favor the presence of disulfide bonds because the oxidized cysteine residues exhibit a distinctive location relative to the transmembrane domains of Tim17 and Tim22 proteins. The cysteine oxidation-based mechanism for Tim17 and Tim22 is widely present among eukaryotes with the increasing complexity for the Tim22 protein from yeast to humans. Our study suggests an important structural role for this covalent modification during the biogenesis of the Tim17 and Tim22 proteins. The requirement for oxidation correlates with the weak hydrophobicity profile for some of the transmembrane domains. Thus, disulfide bond formation could support the proper positioning of weak transmembrane domains in the bilayer of inner mitochondrial membrane assisting in the assembly of mitochondrial membrane translocase complexes.

## Methods

### Yeast strains and plasmid construction

The *Saccharomyces cerevisiae* strains are derivatives of the YPH499 (*MATa, ade2-101, his3-Δ200, leu2-Δ1, ura3-52, trp1-Δ63, lys2-801*). The temperature-sensitive *tim17-4int* (83) *mia40-4* (YPH-fomp2-7; 176), *mia40-3* (YPH-BGfomp2-8; 178), *mia40-*F311E (660) and *erv1-5* (YPH-BGErv1ts-C159S; 318) as well as Tim18_protA_ (574) were described previously 5,16,18,24,27. The *tim22* cysteine mutant strains with a wildtype version of Tim22 (640) or Tim22-C42S (641), Tim22-C141S (642), Tim22-C42,141S (643) were described previously^16^. The wildtype *C. albicans* strain was DAY185 (*URA3*^+^ *ARG4*^+^ *HIS1*^+^). The *S. cerevisiae TIM17* and *TIM22* genes were amplified, using primers encoding additional extension with Myc or 6xHis tag, from the genomic DNA as a template and cloned into the pESC-URA under GAL inducible promoter (Agilent). This procedure give rise to pLIWHisTIM17 (453p), pLIWMycTIM22 (459p), pLIWMycTIM17 (458p), pMChTIM22-His (206p). Mutated versions of *TIM17* were generated by site-directed mutagenesis using pLIWHisTIM17 (453p) resulting in generation of pLIWHisTIM17-C10S (454p), pLIWHisTIM17-C77S (455p), pLIWHisTIM17-C118S (456p) and pLIWHisTIM17-C120S (457p). *C. albicans TIM17* and *TIM22* genes were amplified from *C. albicans* genomic DNA as a template and cloned into the *pTNT Vector (Promega),* resulting in the generation of pTIM17CaWT (120p) and pTIM22CaWT (121p). Cysteine mutant versions of *C. albicans* Tim22 were generated by site-directed mutagenesis, resulting in the generation of pTIM22Ca-C118S (122p), pTIM22Ca-C156S (123p), and pTIM22Ca-C118,156S (124p). An additional cysteine residue in *S. cerevisiae* Tim22 at position 195 of the amino acid sequence was introduced using a reverse primer, resulting in the generation of the Tim22-S195C PCR product. *TIMM22* cDNA was obtained from a HeLa cDNA library using specific primers that introduced the His-tag on the N-termini of the protein. The tagged version of TIMM22 was cloned into a pcDNA3.1 Zeo+ vector, thus generating _His_TIMM22-WT (pAS_hTimm22-Nhis; 393p). Cysteine mutants of human TIMM22 were generated by site-directed mutagenesis, resulting in the generation of _His_TIMM22-C69S (pAS_hTimm22C69S-Nhis; 394p), _His_TIMM22-C138S (pAS_hTimm22C138S-Nhis; 396p), and _His_TIMM22-C160S (pAS_hTimm22C160S-Nhis; 397p).

### Growth of yeast and mitochondria isolation

Yeast were grown on liquid or solid YPG (1% [w/v] yeast extract, 2% [w/v] bactopeptone, and 3% [w/v] glycerol) or YPD (1% [w/v] yeast extract, 2% [w/v] bactopeptone, and 2% [w/v] glucose) medium. To induce the *GAL10* promoter for the expression of Myc- and His- tag fusion proteins, yeast were transformed with plasmids and grown on a selective medium (0.67% [w/v] yeast nitrogen base, 0.079% [w/v] CSM amino acid mix and 3% [w/v] glycerol) with addition of 0.5% [w/v] galactose. The yeast strains were grown at the permissive temperature 19 °C–24 °C or were shifted for 6 h to the restrictive temperature 37 °C. Mitochondria were isolated by differential centrifugation according to the standard procedures^28^ and were resuspended in SM buffer (250 mM sucrose and 10 mM MOPS-KOH [pH 7.2]).

### Human cell culture and transfection

HEK293 and MCF-7 cells were cultured at 37 °C in a 5% CO_2_ atmosphere. The cell lines were sustained in Dulbecco’s modified Eagle’s medium (DMEM) with high glucose content (4500 mg/L), supplemented with 10% (v/v) heat-inactivated (HEK293) or non heat-inactivated (MCF-7) fetal bovine serum (FBS), 2 mM L-glutamine, and 1% (v/v) penicillin-streptomycin. For the overexpression of TIMM22 constructs, 2 × 10^6 ^cells were seeded on 10-cm dishes 48 h before transfection. The cells were plated in high-glucose (4500 mg/L) DMEM medium. After 24 h, the media was changed to low-glucose (1000 mg/L) DMEM medium, and cells were cultured for a further 24 h. Next, the media was changed to galactose (10 mM) DMEM medium, and the cells were transfected with pcDNA3.1 Zeo+-containing _His_TIMM22-WT, _His_TIMM22-C69S, _His_TIMM22-C138S, and _His_TIMM22-C160S per plate using polyethylenimine or JetPrime (Polyplus-transfections). Cells were harvested 24 h after transfection.

### Mitochondria isolation from human cells

Cells were plated in low-glucose (1000 mg/L) DMEM medium and cultured until the glucose level was below detection (around 24 h). Next, the media was changed to DMEM that contained galactose (10 mM), and the cells were cultured for a further 24 h. The cells were collected by scraping and homogenized using a Dounce homogenizer in isolation buffer (220 mM mannitol, 70 mM sucrose, 1 mM ethylenediaminetetraacetic acid [EDTA], 2 mM phenylmethanesulfonyl fluoride [PMSF], 20 mM Hepes/KOH [pH 7.6], and 2 mg/ml bovine serum albumin [BSA]). The supernatant was centrifuged twice at 1,500 × *g* for 5 min to remove cellular debris. The crude mitochondrial pellet was obtained by centrifugation at 14,000 × *g* for 15 min at 4 °C and resuspended in isolation buffer without BSA. Protein content was quantified using the Bradford assay, and the mitochondrial pellet of the desired concentration was obtained by centrifugation at 14,000 × *g* for 15 min at 4 °C. For fractionation, the cells were resuspended in isolation buffer without BSA and incubated for 10 min on ice prior to homogenization. The samples were centrifuged at 1,500 × *g* for 5 min, and the supernatant was divided into three equal volumes, representing total (T), post-mitochondrial supernatant (S), and mitochondrial (M) fractions. The T and S fractions were precipitated with 10% trichloroacetic acid (TCA), and the M fraction was centrifuged at 14,000 × *g* for 15 min at 4 °C. The pellets were resuspended in Laemmli buffer with 50 mM dithiothreitol (DTT) and incubated for 15 min at 65 °C.

### Thiol modification assays in yeast

Isolated yeast mitochondria were solubilised in digitonin-containing buffer (1% [w/v] digitonin, 20 mM Tris-HCl [pH 7.4], 100 mM NaCl, and 1 mM PMSF) in the presence or absence of the reducing agent TCEP for 20 min on ice. For modification with mPEG_5_ (63187, Sigma-Aldrich), mitochondria were incubated in lysis buffer (6 M Urea, 0.5% sodium dodecyl sulfate [SDS], 200 mM Tris-HCl [pH 7.4], and 10 mM EDTA [pH 8.0]) as indicated prior to mPEG modification. The samples were precipitated with 10% TCA for 30 min on ice and centrifuged at 20,000 × *g* for 15 min at 4 °C. The pellets were washed with ice-cold acetone, dried, and resuspended in Laemmli buffer that contained 10 mM AMS, 50 mM IAA or 50 mM DTT. The samples were incubated for 30 min at 30 °C and denatured for 15 min at 65 °C, and proteins were separated on SDS-polyacrylamide gel electrophoresis (PAGE) followed by Western blot. Primary antibodies were custom-raised in rabbits and controlled for specificity. Specific protein bands were detected using secondary anti-rabbit antibodies conjugated with horseradish peroxidase (Sigma-Aldrich) and chemiluminescence.

### Thiol trapping assay in human cells

For the direct thiol trapping assay, free thiol groups were directly modified by suspending the mitochondrial pellet in incubation buffer A (0.5% SDS, 6 M Urea, and 0.2 M Tris-HCl [pH 6.8]). In the case of TIMM22 variants, the mitochondrial pellet was suspended in buffer B (1% n-Dodecyl β-D-maltoside [DDM], 1% SDS, and 20 mM Tris-HCl [pH 7.4]) that contained 15 mM AMS or 10 mM mPEG_1.2_ (22713, Thermo Scientific). As a control, the existing disulfide bonds were reduced with 100 mM DTT. The samples were incubated for 30 min at 30 °C. Proteins were precipitated by the addition of 10% TCA or acetone. For the indirect thiol trapping assay, free thiol groups were modified by incubating mitochondria in buffer A that contained either 50 mM IAA or 15 mM AMS. The samples were incubated for 30 min at 30 °C, followed by 30 min at 65 °C. The samples were then diluted with water to reach a 1.6 M urea concentration, and the proteins were precipitated by the addition of 5× the volume of acetone and kept overnight at −20 °C. Proteins were collected by centrifugation at 4,500 × *g* for 30 min at 4 °C and washed with cold acetone. The pellets were resuspended in buffer A, and disulfide bonds were reduced by the addition 10 mM TCEP. The samples were then incubated for 30 min at 30 °C, followed by 30 min at 65 °C. The proteins were precipitated as described previously, and the pellets were resuspended in buffer A that contained 15 mM AMS to modify free cysteine residues that arose and incubated for 30 min at 30 °C, followed by incubation for 30 min at 65 °C. Proteins were precipitated, and pellets were resuspended in Laemmli buffer with 100 mM DTT and incubated for 15 min at 65 °C, followed by separation on SDS-PAGE and Western blot. Commercially available antibodies for rabbit polyclonal anti-TIMM17B (11062-1-AP; Protein Tech), anti-TIMM22 (14927-1-AP; Protein Tech), anti-MIC60 (NB100-1919, Novus Biologicals), anti-TOMM20 (sc-11415, Santa Cruz), anti-MIC19 (HPA042935; Atlas Antibodies) and anti-CYCS (ab133505, Abcam) and mouse monoclonal anti-β-actin (A1978; Sigma-Aldrich), anti-ATP5A (ab14748; Abcam), anti-TIMM23 (611222; BD Biosciences), were used.

### *In vitro* import of radiolabeled precursor proteins

Radiolabeled precursors were synthesized in rabbit reticulocyte lysate (Promega) with the addition of [^35^S]methionine (Perkin Elmer). The import of radiolabeled precursor proteins into isolated *S. cerevisiae* or *C. albicans* mitochondria was performed at 25 °C or 30 °C in import buffer (250 mM sucrose, 80 mM KCl, 5 mM MgCl_2_, 5 mM methionine, 10 mM MOPS-KOH, 10 mM KH_2_PO_4_ [pH 7.2], 3% BSA, 2 mM ATP, and 2 mM NADH). The import reaction was stopped by the addition of VOA mixture (1 μM valinomycine, 20 μM oligomycine, and 8 μM antimycin A), 50 mM IAA, or 10 mM AMS. To remove excess non-imported precursors, the mitochondria were treated with 50 μg/ml proteinase K for 15 min on ice. To inhibit the activity of proteinase K, the samples were treated with 2 mM PMSF. The mitochondria were centrifuged at 20,000 × *g* for 15 min at 4 °C, and the pellet was washed with SM buffer. The samples were incubated in Laemmli buffer with 50 mM IAA (non-reducing conditions) or 50 mM DTT (reducing conditions) at 65 °C for 15 min and analyzed by SDS-PAGE. For blue native analysis of protein complexes, mitochondria were resuspended in digitonin solubilization buffer (1% [w/v] digitonin, 20 mM Tris-HCl, pH 7.4, 50 mM NaCl, 10% [w/v] glycerol, 0.1 mM EDTA, 1 mM PMSF). Soluble fraction was separated on a 6–16.5% gradient gel at 4 °C. Radiolabeled proteins were detected by digital autoradiography (Typhoon Trio, GE Healthcare) and processed using ImageQuant (GE Healthcare).

### Affinity purification

Affinity purification via Tim18_ProtA_ was performed as previously described^16^. For affinity purification via _His_TIMM22, 1 mg of HEK293 cells was solubilised in digitonin buffer (1% [w/v] digitonin, 10% [w/v] glycerol, 20 mM Tris-HCl [pH 7.4], 100 mM NaCl, 20 mM imidazole, 50 mM IAA, and 1 mM PMSF) for 20 min on ice. Solubilised material was clarified by centrifugation at 20,000 × *g* for 15 min at 4 °C, and the supernatants were incubated with Ni-NTA (Qiagen) for 1 h at 4 °C. Ni-NTA was washed twice with washing buffer (20 mM Tris-HCl [pH 7.4], 50–100 mM NaCl, and 20 mM imidazole). Bound proteins were eluted by incubation with elution buffer (20 mM Tris-HCl [pH 7.4], 100 mM NaCl, and 400 mM imidazole). Eluted proteins were precipitated with StrataClean resin (Agilent Technologies). The samples were incubated in Laemmli buffer with 50 mM DTT at 65 °C for 15 min and analyzed by SDS-PAGE followed by Western blot.

### Carbonate extraction and protease accessibility assay

Isolated mitochondria (typically 200 μg) were resuspended in 420 μl of ice-cold 0.1 M Na_2_CO_3_, pH 10.8, and incubated for 30 min on ice. The samples were divided into a total fraction (T) and a fraction that was ultracentrifuged at 125,000 × *g* for 1 h at 4 °C. The supernatant fraction (S) was collected in a new tube. The pellet fraction (P) was resuspended in Laemmli buffer that contained 50 mM DTT. The T and S fractions were precipitated with 10% TCA for 30 min on ice, centrifuged at 20,000 × *g* for 15 min at 4 °C, and washed with ice-cold acetone. For the protease accessibility assay, isolated mitochondria were incubated in SM buffer (250 mM sucrose and 10 mM MOPS-KOH, pH 7.2) or M buffer (10 mM MOPS/KOH, pH 7.2) on ice for 30 min to generate mitoplasts by hypo-osmotic swelling. Half of each sample was treated with indicated concentration of proteinase K for 15 min. The activity of proteinase K was inhibited by addition of 3 mM PMSF. The samples were centrifuged at 20,000 × g for 15 min at 4 °C. The pellets were resuspended in Laemmli buffer that contained 50 mM DTT. The samples were denatured for 15 min at 65 °C and analyzed by SDS-PAGE followed by Western blot. For detection of tagged proteins the antibodies anti-Myc (05–724; Milipore) and anti-His (34660; Qiagen) were used.

### Sequence analyses

Protein sequences were obtained from UniProt database. Multiple sequence alignment was performed using Clustal Omega (EMBL-EBI)^29^. Output alignments were further processed in Adobe Illustrator to highlight conserved cysteine residues and transmembrane domains. The cysteine residues were marked with red boxes. The highly conserved amino acid residues were marked in yellow. Transmembrane topology predictions were performed using hmmtop^30^, Phobius^31^, TMPred (Expasy).

## Additional Information

**How to cite this article**: Wrobel, L. *et al.* The presence of disulfide bonds reveals an evolutionarily conserved mechanism involved in mitochondrial protein translocase assembly. *Sci. Rep.* **6**, 27484; doi: 10.1038/srep27484 (2016).

## Supplementary Material

Supplementary Information
