# Supplementary material for: The presence of disulfide bonds reveals an evolutionarily conserved mechanism involved in mitochondrial protein translocase assembly
Source: Sci Rep. 2016 Jun 6;6:27484. doi: 10.1038/srep27484 (PMC4893733; doi:10.1038/srep27484)
Supplement: Supplementary Information [file srep27484-s1.pdf]

Supplementary Information

The presence of disulfide bonds reveals an evolutionarily conserved mechanism involved in mitochondrial protein translocase assembly

Lidia Wrobel,[1,2] Anna M. Sokol,[1] Magdalena Chojnacka,[1] and Agnieszka Chacinska,[1,*]

[1]International Institute of Molecular and Cell Biology, 02-109 Warsaw, Poland

[2] Department of Microbiology, Monash University, Melbourne, Victoria 3800, Australia

*Corresponding author: achacinska@iimcb.gov.pl;

Supplementary Figure Legends

Supplementary Figure S1. Import and oxidation of Tim17. (a) Radiolabeled *S. cerevisiae* Tim17 precursors were incubated with mitochondria that were isolated from wildtype *S. cerevisiae*. The import reaction was stopped by transferring the samples on ice and adding 10 mM AMS. (b) Wild-type and *tim17-4int* strains were subjected to consecutive 10-fold dilutions, spotted on YPD and YPG, and grown for 3–7 d at the indicated temperatures. (c) Mitochondria isolated from strain expressing $_{Myc}$Tim17 were subjected to alkaline carbonate extraction. T, total mitochondrial protein extract; S, supernatant; P, pellet of mitochondrial membranes. (d) Mitochondria isolated from strain expressing $_{Myc}$Tim17 were incubated in hypotonic buffer for 30 minutes to generate mitoplasts, when indicated. Mitoplasts and intact mitochondria were treated with 50 µg/ml proteinase K, when indicated. (a, c, d) The samples were analyzed under reducing or non-reducing conditions by SDS-PAGE followed by Western blot or autoradiography. ox, oxidized; WT, wildtype.

Supplementary Figure S2. Alignment of Tim17. Conservation of two cysteine residues in Tim17 revealed by multiple sequence alignment, indicated by the red asteriscs. *Sc*, *S. cerevisiae*; *Sp*, *Schizosaccharomyces pombe*; *Nc*, *Neurospora crassa*; *Ca*, *Candida albicans*; *Ce*, Caenorhabditis *elegans*; *Dm*, *Drosophila melanogaster; Xl, Xenopus laevis*; *Hs-A, Homo sapiens isoform A; Hs-B, Homo sapiens isoform B.*

Supplementary Figure S3. Import analyses of membrane Tim proteins. (a) Mitochondria were isolated from wildtype *S. cerevisiae* strain and pretreated with TCEP prior to denaturation in Laemmli buffer with 50 mM IAA or 10 mM AMS. (b) Mitochondria were

isolated from HEK293 cells and treated either with 100 mM DTT (lane 1) or with 15 mM AMS (lane 2). An indirect thiol trapping assay was performed to identify the presence of disulfide bonds. Free cysteine residues were modified by 50 mM IAA or 15 mM AMS (lanes 3 and 4), and disulfide bonds were then reduced with 10 mM TCEP to yield free cysteine residues that were further modified with 15 mM AMS (lanes 3 and 4). *(c)* Radiolabeled Tim17 precursor was incubated with mitochondria isolated from wildtype and *erv1-5* mutant strains grown at 19°C. Nonimported protein was removed by proteinase K and samples were analysed under non-reducing conditions in the presence of 10 mM AMS. Right panel: quantification of radiolabeled Tim17 import. (d) Radiolabeled Tim22 wildtype or Tim22-S195C precursor was imported into isolated mitochondria, followed by proteinase K digestion of nonimported precursors. The mitochondria were further incubated with 2 mM $mPEG_5$ for 30 min at 25°C. Mitochondria were subjected to alkaline carbonate extraction. T, total protein extract; S, supernatant; P, membrane pellet. (a-d) Samples were analyzed by SDS-PAGE followed by Western blot or autoradiography. ΔΨ, inner membrane electrochemical potential; WT, wildtype.

Supplementary Figure S4. Tim22 sequence alignment. (a) Conservation of cysteine residues in fungi Tim22 revealed by multiple sequence alignment, indicated by the red asteriscs. *Sc*, *S. cerevisiae*; *Sp*, *S. pombe*; *Nc*, *N. crassa*; *Cg*, *Candida glabrata*; *Ca*, *C. albicans*; *Af, Aspergillus flavus*. (b) High conservation of six cysteine residues revealed by multiple sequence alignment of metazoan Tim22. *Dr, Danio rerio; Xl, Xenopus laevis; Mm, Mus musculus; Bs, Bos taurus; Hs, Homo sapiens.*

Supplementary Figure S5. Characterization of *S. cerevisiae tim22* cysteine mutants. (a) Wildtype and *tim22* cysteine mutant strains were subjected to consecutive 10-fold dilutions, spotted on the minimal medium with addition of 2% glucose or 3% glycerol, and grown at the indicated temperatures. (b) Mitochondria were isolated from wildtype and *tim22* cysteine mutant strains grown on YPG liquid medium at 19°C and shifted for 6 h to 37°C. Mitochondrial proteins were analysed by SDS-PAGE followed by Western blot. (c) Radiolabeled wildtype Tim22 and *tim22* cysteine mutant precursors were incubated with wildtype mitochondria. Mitochondria were solubilised in digitonin-containing buffer and analyzed by blue native electrophoresis. ΔΨ, inner membrane electrochemical potential; WT, wildtype.

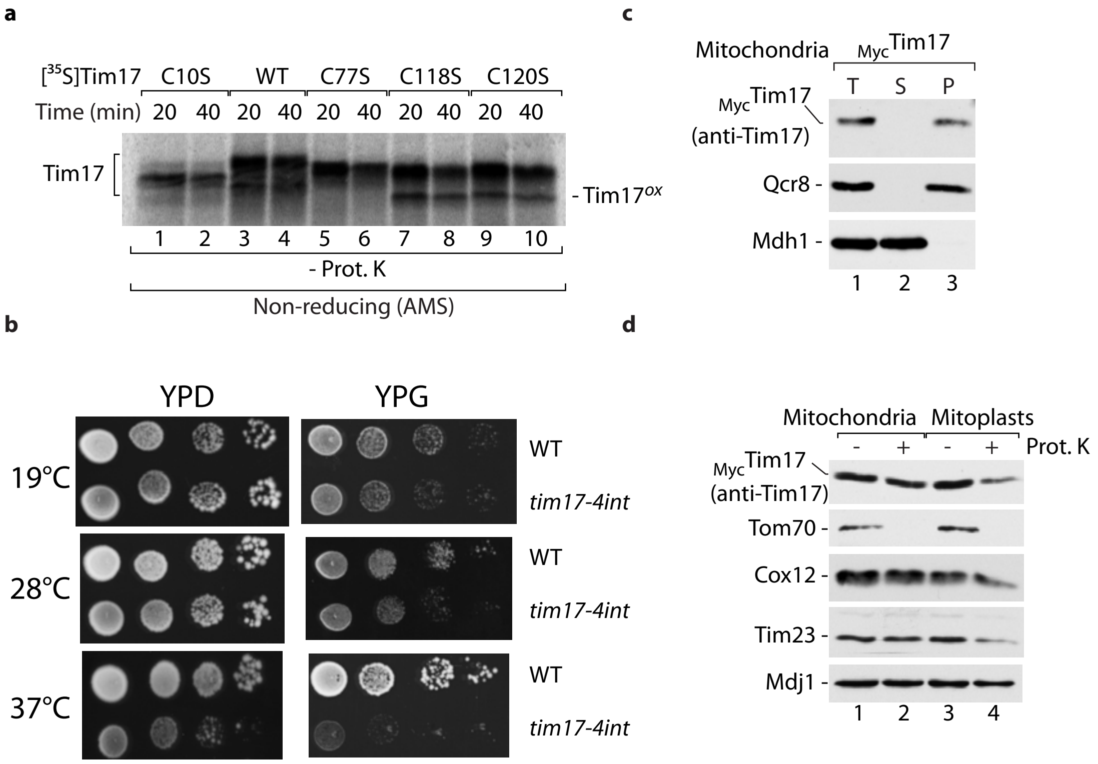

Figure S1 Wrobel et al

Tim17 alignment

```
                       *      TM1
Sc      -MSADHSRDPCPIVILNDFGGAFAMGAIGGVVWHGIKGFRNSPLG--ERGSGAMSAIKAR  57
Sp      MASADHTRDPCPYVILNDFGAAFSMGTIGGAIWHSIKGWRNSPPG--EKRISAIAAAKTR  58
Nc      ---MDHTRDPCPWVILNDFGGAFAMGAIGGTIWHGIKGFRNSPYG--ERRIGAITAIKMR  55
Ca      -MSADHTRDPCPIVILNDFGGAFAMGVIGGCVWHGIKGFRNSPYG--ERSYGALSAIKAR  57
Ce      --MEEYTREPCPYRIGDDIGSAFAMGLVGGSIFQAFGGYKNAAKG--KKLVGMMREVRMR  56
Dm      --MEEYAREPCPYRIVDDCGGAFAMGCIGGGVFQAIKGFRNAPSGLNRRLVGSIIAIKTR  58
Xl      --MEEYMREPCPWRIVDDCGGAFTMGIIGGGVFQAVKGFRNAPAGVGHRLRGSMSAVRIR  58
Hs-A    --MEEYAREPCPWRIVDDCGGAFTMGTIGGGIFQAIKGFRNSPVGVNHRLRGSLTAIKTR  58
Hs-B    --MEEYAREPCPWRIVDDCGGAFTMGVIGGGVFQAIKGFRNAPVGIRHRLRGSANAVRIR  58

              TM2        *              TM3                 TM4
Sc      APVLGGNFGVWGGLFSTFDCAVKAVRK-REDPWNAIIAGFFTGGALAVRGGWRHTRNSSI  116
Sp      APVLGGNFGVWGGLFSTFDCAVKGVRR-KEDPWNAIIAGFFTGGALAVRGGWRATRNGAI  117
Nc      APALGGNFGVWGGLFSTFDCAIKGLRNHKEDPWNSILAGFFTGGALAVRGGYKAARNGAI  115
Ca      APVVGGNFGVWGGLFSTFDCTVKAVRK-REDAWNAVIAGFFTGGALAIRGGWKHTRNSAI  116
Ce      STLTGVQFAAWGGMFSTIDCCLVAIRK-KEDPINSIVSGGLTGALLAIRSGPKVMAGSAI  115
Dm      SPVIAGNFAVWGGMFSTIDCTLVHFRK-KEDPWNSIISGAATGGILAARNGVPAMAGSAI  117
Xl      APQIGGSFAVWGGLFSTIDCGLVRLRG-KEDPWNSITSGALTGAVLASRSGPLAMVGSAL  117
Hs-A    APQLGGSFAVWGGLFSMIDCSMVQVRG-KEDPWNSITSGALTGAILAARNGPVAMVGSAA  117
Hs-B    APQIGGSFAVWGGLFSTIDCGLVRLRG-KEDPWNSITSGALTGAVLAARSGPLAMVGSAM  117

            TM4
Sc      TCACLLGVIEGVGLMFQRYAAWQAKPMAPPLPE--APSSQPLQA----------------  158
Sp      GCACILAVFEGLGIALGRMNAEYNRPVAPVIPD--APASGSTSA-APAAV----------  164
Nc      GCAVLLAVIEGVGIGFQKMLAGATKLEAPAPPP--SNEKVLA------------------  155
Ca      TCACLLGVFEGVGMMMQRLQAQP--TMAPVYPE--EPTALAA------------------  154
Ce      LGSVILAMIEGVGLVTTRWMGAMMDPTQPPPEALDDPRSLGQKSQA--EPGLDQTRPFGI  173
Dm      IGGVLLALIEGVGILFTRISADQFKNPIPP---AEDPVALGDPGRNFSFESASNRTQYQ-  173
Xl      MGGILLALIEGVGILLTRYTAQQFQNPNPF---GEDTSHMTH------------------  156
Hs-A    MGGILLALIEGAGILLTRFASAQFPNGPQF---AEDPSQLPSTQ-LP-SSPFGDYRQYQ-  171
Hs-B    MGGILLALIEGVGILLTRYTAQQFRNAPPF---LEDPSQLPPKDGTP-APGYPSYQQYH-  172

Sc      --------   158
Sp      --------   164
Nc      --------   155
Ca      --------   154
Ce      PTGLPNLS   181
Dm      --------   173
Xl      --------   156
Hs-A    --------   171
Hs-B    --------   172
```

Figure S2 Wrobel et al

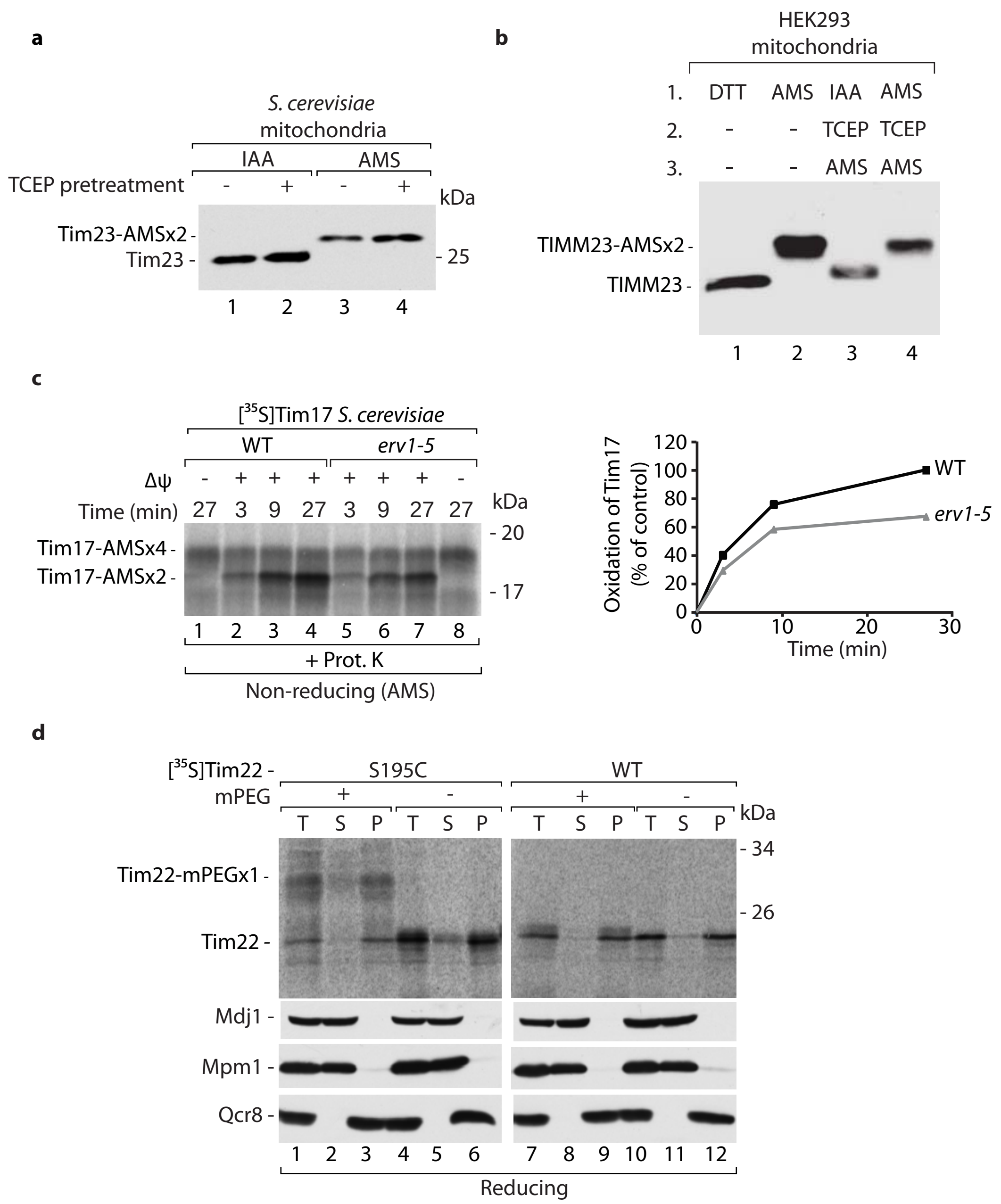

Figure S3 Wrobel et al

a

Tim22 alignment (Fungi)

```
                                                               *     TM1
Sc   MVYTGFGLEQISPAQK---------KPYNELTPEEQG----ERGAEMIMNFMTSCPGKSV   47
Sp   -----MSLSGLLPPNLGGNS----PNADGNLTPEEKAALQQAKIIGYMNRISESCVFKSS   51
Nc   MNFP--GMPGGAAPSGGAAPGGYD-----------PN----DPNIKMMQKAMESCFAKTV   43
Cg   MVYRGFGLEYLSPPEK---------KAFGELSPDEQG----ERGAEMVVGFMSSCPGKSV   47
Ca   --MSLWGVYTGPQPPK---------KPLQEMTQEEQA----EEGARQMIGFMNSCPGKTV   45
Af   MNIP--GMTTGMAPAGATAGAGFPGAGAGMQGMSEQE----QAMVKAMHAAMESCPVKTV   54

           TM1
Sc   VSGVTGFALGGVLGLFMASMAYDTPLHTPTPANTAATATAGNIGVGGISRTVQQISDLPF   107
Sp   MAGVLGFGLGGIFGMFISSLDLQH-------------------------IDPKIYEKPF   85
Nc   MSGGAGFALGGVFGMFMASMAYDTPYHSPTTPGTGPGANPAAAGIPGY--KPVDLSSMPL   101
Cg   ISGATGFALGGVLGLFMASMAYDTPLHTPVPG--------------GMSGAVQQMADLPL   93
Ca   MAGVSGFALGGFFGLFMASMAYDTPIGTD--------------------AVKHISELPF   84
Af   ISGTMGFGLGGVFGLFMASMSYDSTFTPQG---------------------KAIMDLPW   92

                              TM2    *              TM3
Sc   RQQMKLQFTDMGKKSYSSAKNFGYIGMIYAGVECVIESLRAKNDIYNGVTAGFFTGAGLA   167
Sp   REQIRIQARDMGSRSFSTAKNFGLLGLIYSGSECCIEAFRAKTDIYNAIAAGVFTGGALA   145
Nc   KEQLKHGFKDMGQRSYSTAKNFAKVGALFSGIECGIEGLRAKNDLGNGVAAGCLTGAILA   161
Cg   RQQVKLQFADMGKRAYSSAKNFGYIGMIYAGVECAVESLRAKNDIYNGITAGCITGGGLA   153
Ca   KQQMKLQFTDMAKRSYSSAKNFGYIGMVYSGVECTIESLRAKHDIYNGVSAGCITGAGLA   144
Af   REQVRRGFKDMGSRSWSSAKNFGIVGALYSGTECCVEGLRAKNDLSNSVISGCITGGILG   152

TM3          TM4
Sc   YKAGPQAALMGGAGFAAFSAAIDLYMKSEDGRPPQNDFKE      207
Sp   VRSGPKAIVLGGAGFGLFSYGIEKYMHWGE----------      175
Nc   KNGGPQAAAVGCAGFAAFSAAIDAWMRMPSEED-------      194
Cg   YKSGPQAALVGCAGFAAFSAAIDMYMKSEDGRPPENDFKQ      193
Ca   IKAGPQAALVGCAGFAAFSLAIDMYLNSDAAPPPKNDYDI      184
Af   AKAGPQAAAAGCAGFAAFSAAIDAYMRMPSEE--------      184
```

b

Tim22 alignment (Higher Eukaryotes)

```
Dr   MAAPAKSTDASLSVTAGHSANVSSAENVSLQYSLILDHLIGDKRQIKDLNPTVMGALPSP   60
Xl   -----------------MGSNVTPPGDGTLQYSLIMQHLVGDKRRPVELIPGGLGGIPTP   43
Mm   -------MAATAPKAGGSAPEAAGSAEAPLQYSLLLQYLVGDKRQPRLLEPGSLGGIPSP   53
Bt   -------MAATAPKAGGSAPEAAASAEAPLQYSLLLQYLVGDKRQPRLLEPGSLGGIPSP   53
Hs   -------MAAAAPNAGGSAPETAGSAEAPLQYSLLLQYLVGDKRQPRLLEPGSLGGIPSP   53

                   *       *    TM1
Dr   QKTDEQKMIERGMESCAFKSLIACVGGFVLGGAFGVFTAGIDANVGLDPKDPLRTPTARE   120
Xl   IKPEEQKMMERVMESCGFKAALACVGGFVLGGAFGVFTAGIDTNVGFDPKDPLRTPTAKE   103
Mm   AKSEEQKMIERAMESCAFKAVLACVGGFVLGGAFGIFTAGIDTNVGFDPKDPYRTPTAKE   113
Bt   AKSEEQKMIERAMESCAFKAALACVGGFVLGGAFGVFTAGIDTNVGFDPKDPYRTPTARE   113
Hs   AKSEEQKMIEKAMESCAFKAALACVGGFVLGGAFGVFTAGIDTNVGFDPKDPYRTPTAKE   113

                       TM2 *  *             TM3 *
Dr   VLKDMGQRGMSYAKNFAIVGAMFSCTECLIESHRGKSDWKNAVYSGCITGGAIGFRAGLK   180
Xl   VLRDMGQRGMSYAKNFAIVGAMFSCTECLVESYRGKSDWKNSVMSGCITGGAIGFRAGLK   163
Mm   VLKDMGQRGMSYAKNFAIVGAMFSCTECLVESYRGKSDWKNSVISGCITGGAIGFRAGVK   173
Bt   VLKDMGQRGMSYAKNFAIVGAMFSCTECLVESYRGKSDWKNSVISGCITGGAIGFRAGLK   173
Hs   VLKDMGQRGMSYAKNFAIVGAMFSCTECLIESYRGTSDWKNSVISGCITGGAIGFRAGLK   173

          *    TM4
Dr   AGVLGCGGFAAFSAAIEYYLR 201
Xl   AGVLGCGGFAAFSAVIDYYLR 184
Mm   AGAIGCGGFAAFSAAIDYYLR 194
Bt   AGVIGCGGFAAFSAAIDYYLR 194
Hs   AGAIGCGGFAAFSAAIDYYLR 194
```

Figure S4 Wrobel et al

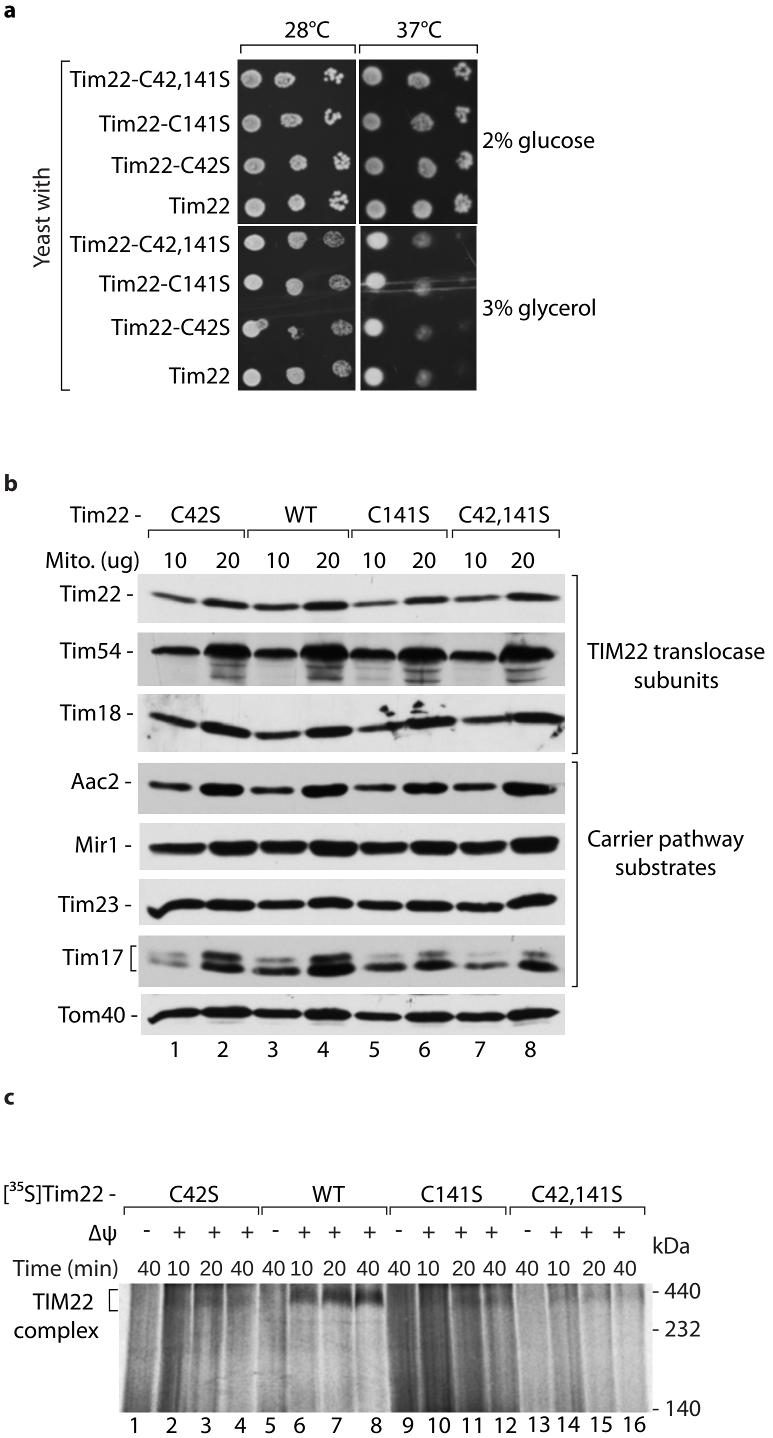

Figure S5 Wrobel et al
